# An Investigation about Gene Modules Associated with hDPSC Differentiation for Adolescents

**DOI:** 10.1155/2019/8913287

**Published:** 2019-04-04

**Authors:** Wenjing Xu, Jianqiang Li, Juan Li, Ji-Jiang Yang, Qing Wang, Bo Liu, Weiliang Qiu

**Affiliations:** ^1^Faculty of Information Technology, Beijing University of Technology, Beijing, China; ^2^Beijing Engineering Research Center for IoT Software and Systems, Beijing University of Technology, Beijing, China; ^3^Tsinghua National Laboratory for Information Science and Technology, Tsinghua University, Beijing, China; ^4^Channing Division of Network Medicine, Brigham and Women's Hospital/Harvard Medical School, Boston, USA

## Abstract

Dental pulp stem cells (DPSCs) have the property of self-renewal and multidirectional differentiation so that they have the potential for future regenerative therapy of various diseases. The latest breakthrough in the biology of stem cells and the development of regenerative biology provides an effective strategy for regenerative therapy. However, in the medium promoting differentiation during long-term passage, DPSCs would lose their differentiation capability. Some efforts have been made to find genes influencing human DPSC (hDPSC) differentiation based on hDPSCs isolated from adults. However, hDPSC differentiation is a very complex process, which involves multiple genes and multielement interactions. The purpose of this study is to detect sets of correlated genes (i.e., gene modules) that are associated to hDPSC differentiation at the crown-completed stage of the third molars, by using weighted gene coexpression network analysis (WGCNA). Based on the gene expression dataset GSE10444 from Gene Expression Omnibus (GEO), we identified two significant gene modules: yellow module (742 genes) and salmon module (9 genes). The WEB-based Gene SeT AnaLysis Toolkit showed that the 742 genes in the yellow module were enriched in 59 KEGG pathways (including Wnt signaling pathway), while the 9 genes in the salmon module were enriched in one KEGG pathway (neurotrophin signaling pathway). There were 660 (7) genes upregulated at P10 and 82 (2) genes downregulated at P10 in the yellow (salmon) module. Our results provide new insights into the differentiation capability of hDPSCs.

## 1. Introduction

Regenerative therapy is to study the repair and regeneration of damaged tissues and organs such as bone regeneration [1]. Hundreds of millions of people need tissue repair and regenerative treatment every year, and regenerative medicine may help these patients to a large extent [2]. The progress in theory and technology and the support of governments all promote a promising future of regenerative therapy [3]. Gronthos et al. identified a type of undifferentiated precursor cells in human dental pulp tissue that can be terminally differentiated into odontoblasts and secrete cell matrices [4]. Like bone marrow stromal stem cells, DPSCs possess the properties of high proliferative potential, the capacity of self-renewal, and multilineage differentiation, which can lead to odontoblast, osteoblast, adipocyte, and nerve cell differentiation [5].

There are two problems to be solved for utilizing stem cells to tissue regeneration and cell therapy: (1) whether stem cells are easy to obtain and (2) whether enough stem cells could be obtained to treat tissue regeneration and repair [6]. DPSCs could help solve these two problems. Obtaining DPSCs is much less painful for patients than obtaining bone marrow stem cells. Moreover, DPSCs are abundant in origin, strong in proliferation, and can be stored at low temperature for long periods of time without loss of differentiation [7].

DPSCs play an important role in tooth regeneration, nerve repair, bone tissue engineering, and translational medicine [8]. With the increase of passage times, fibroblasts grew flourishingly and the number of DPSCs decreased [9]. Takeda et al. confirmed that cells could not produce dentin-like tissue in vivo after 10 passages whereas P4 transplants showed a layer of dentin-like matrix covered with an interface layer of odontoblast-like cells [10]. In order to identify the key genes that affect the differentiation of DPSCs by passage, Takeda et al. [10] performed an experiment, in which they selected six independent samples at the crown-completed stage that had undergone 4 passages (P4) or 10 passages (P10) and examined gene expression levels measured by the Human Genome U133 Plus 2.0 platform (GeneChip; Affymetrix, Santa Clara, CA, USA), which could measure the expression levels of 54,675 gene probes. By comparing gene expression levels at P4 and those at P10, Takeda et al. [10] detected 719 genes having more than 2-fold downregulation at P10 than at P4 and 642 genes having more than 2-fold upregulation at P10 than at P4. Takeda et al. [10] concluded that the expressions of several genes, such as WNT16, were significantly changed with the increase of the number of passages, which may lead to the loss of the differentiation ability of hDPSCs.

However, it is unlikely that complex human traits are associated to a single gene or to a bunch of unrelated genes. Instead, it is believed that the interplay of many genes would play important roles in the development of complex human diseases. In this study, we aimed to identify gene network modules that are associated with the dental passage based on Takeda et al.'s [10] hDPSC experiment; the data of which can be downloaded from Gene Expression Omnibus (GEO accession number: GSE10444) [11].

## 2. Materials and Methods

### 2.1. Expression Data

GEO is the largest and most comprehensive public gene expression data resource today. GSE10444 is a dataset stored gene expression levels obtained from Takeda et al.'s [10] hDPSC experiment. GSE104444 has expression levels of 54,675 gene probes for a total of 6 independent samples. We analyzed on 6 independent samples at the crown-completed stage (DP2, 28, and 31) that had undergone P4 and P10.

### 2.2. Data Cleaning

Gene expression data often need to be preprocessed, including detecting missing values, outlying probes and/or arrays, and technical batches and performing data transformation and normalization [12]. In this study, we did the following data preprocessing. We first excluded 15,079 exemplar probes and 62 control probes. We then removed 50 gene probes with negative expression level and then removed 10,128 probes without annotations of gene symbols or Entrez IDs. We next did log2 transformation of expression to make the distribution of expression levels closer to a normal distribution. We then performed quantile normalization to reduce the effects of technical noises. The plot of quantiles of expression levels across arrays is shown in Supplementary Figure 1. The plot of the first two principal components (Supplementary Figure 2) indicated no obvious batch effects, except for the effect of passage.

We also performed a cluster analysis of the samples to detect whether there was any abnormality in the samples. The dendrogram (Supplementary Figure 3) showed that there are no obvious outliers.

### 2.3. An Overview of Weighted Gene Coexpression Network Analysis (WGCNA)

WGCNA is a systematic biology algorithm for constructing gene coexpression networks [13]. The WGCNA algorithm first assumes that the genetic network is a scale-free network with gene probes as nodes. Two probes are connected by an edge if they are coexpressed. The measure of the coexpression depends on a soft threshold *β* [14]. The value of *β* is chosen so that the network is close to a scale-free network. WGCNA uses hierarchical clustering to detect modules, which are highly connected subnetworks. The coexpression within a module is high, while the scores of different modules of the gene coexpression are low [15]. Finally, WGCNA explores the associations between the modules and a phenotype of interest.

### 2.4. Description of Data Input

WGCNA requires that the users provide three data matrices: (1) gene expression data matrix (rows are gene probes and columns are arrays), (2) feature data matrix (rows are gene probes and columns are feature variables describing probes), and (3) phenotype data matrix (rows are arrays and columns are phenotype variables describing arrays). In the dataset GSE10444, the feature variable “Sequence Type” indicates that there are three types of probes: “Consensus sequence” probes, “Exemplar sequence” probes, and “Control sequence” probes [16]. A Consensus sequence is a nucleotide sequence that is assembled by Affymetrix and is based on one or more sequences from a public database. An Exemplar is a single nucleotide sequence taken directly from a public database. We just used Consensus sequence to do WGCNA.

### 2.5. Association of Gene Modules to a Trait

Two quantities can be used to measure the associations of a gene module to the hDPSC passage. The first one is the module-trait relationships, which is the Pearson correlations between the module eigengenes and the hDPSC passage. The other quantity is the average gene significance (GS) of a gene module. The gene significance of a gene is the -log10(*p* value) of the test for the association of the gene to the hDPSC passage. The average of the GSs for all genes in a gene module is called module significance (MS).

### 2.6. Pathway Enrichment Analysis

Pathway enrichment analysis is important for elucidating the molecular mechanisms of a set of genes. Usually, the genes enriched in the same pathway play similar roles. Pathway databases, especially Kyoto Encyclopedia of Genes and Genomes (KEGG) [17], have been widely used as a reference knowledge base for biomedical scientists to interpret their experimental findings. KEGG represents a database consists of known genes and their respective biochemical functions. The KEGG project is run by the Institute for Chemical Research at Kyoto University, as part of the Japanese Human Genome Program. KEGG consists of the following five main types of data: (1) pathway maps represented by graphical diagrams; (2) ortholog group tables represented by HTML tables; (3) molecular catalogues represented by HTML tables or hierarchical texts; (4) genome maps represented by Java graphics; and (5) gene catalogues represented by hierarchical texts. In this paper, we used WEB-based Gene SeT AnaLysis Toolkit [18] to do KEGG pathway enrichment analysis.

## 3. Results and Discussion

**Figure 1:**
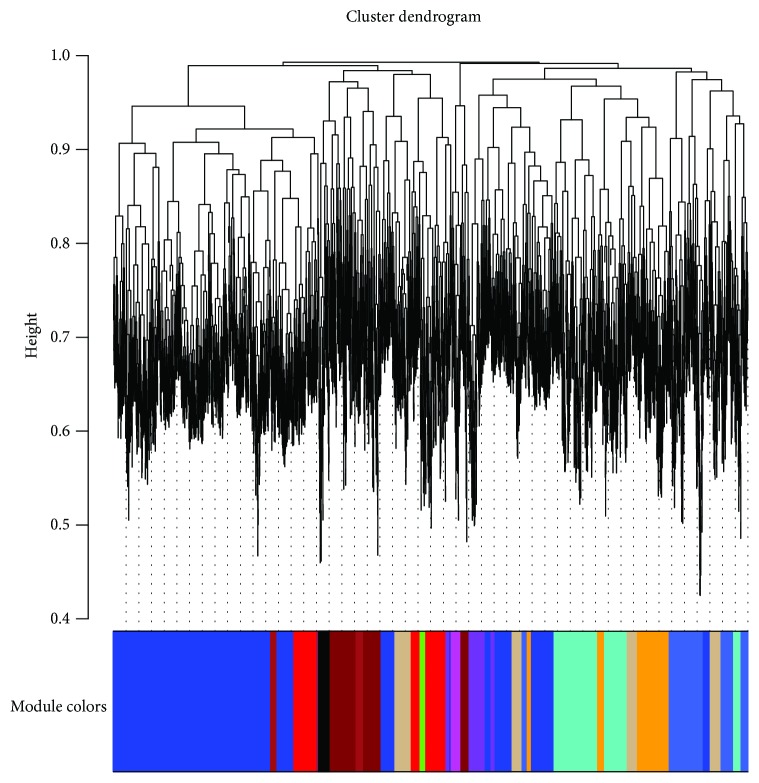
Cluster dendrogram. Dendrogram of the gene modules obtained by hierarchical clustering using adjacency-based dissimilarity. The colored bar below the dendrogram indicates module membership.

**Figure 2:**
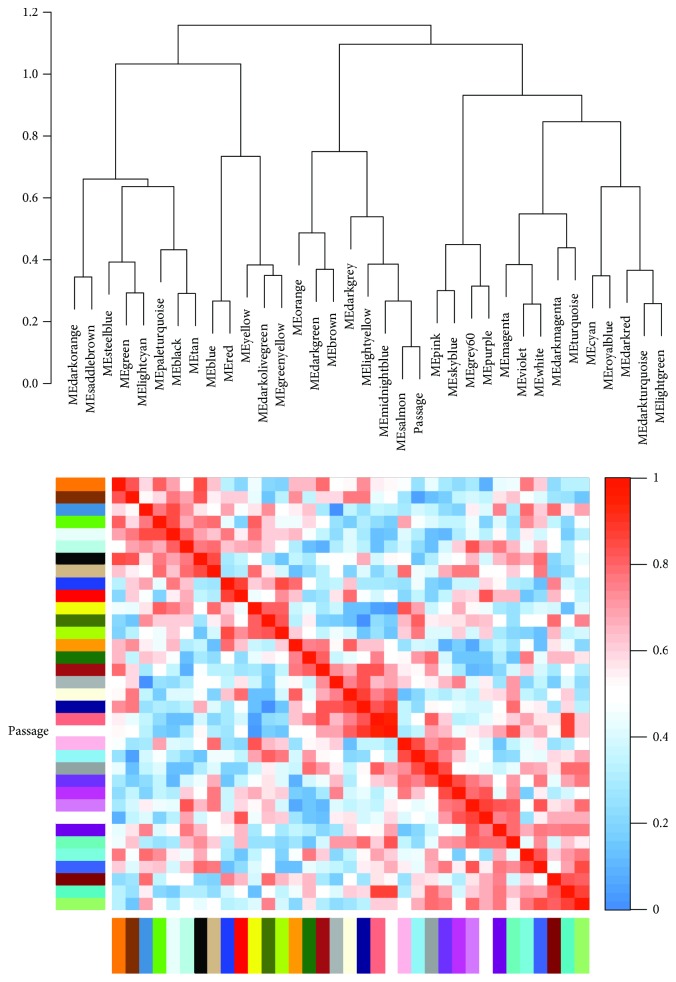
The eigengene dendrogram and eigengene heat map. The top panel shows the dendrogram of the module eigengenes. The bottom panel is the heat map of correlations among the module eigengenes. In the heat map, red color indicates positive correlation, blue color indicates negative correlation, and white color indicates no correlation.

**Figure 3:**
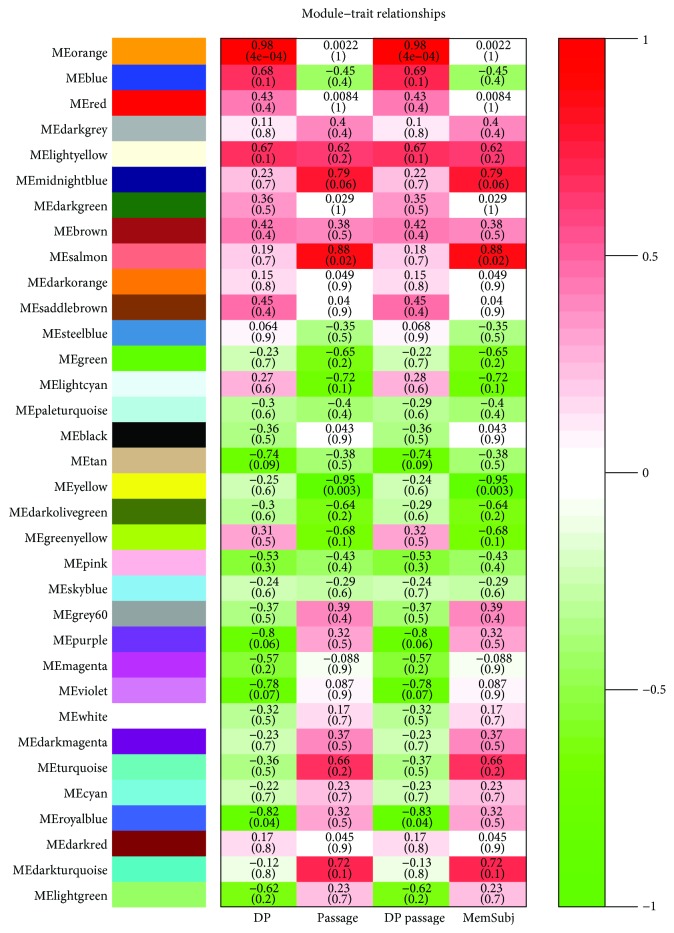
Module-trait relationships. The table is color-coded by correlation. The red color represents a positive correlation between the module eigengene and a trait, the green color represents a negative correlation, and the white color indicates no correlation.

We applied WGCNA to the gene microarray dataset GSE10444 downloaded from GEO to investigate the effect of passage on the differentiation of human dental pulp stem cells (hDPSCs) and to construct gene coexpression modules. Thirty-four modules were identified by WGCNA, ranging from 41 to 742 genes in size (Figure 1). The eigengene dendrogram and eigengene heat map in Figure 2 showed that some module eigengenes are highly correlated with each other.  Figure 3 showed that the yellow module, containing 742 genes, was significantly negatively correlated with hDPSC passage (Pearson correlation = −0.95, *p* value = 0.003), and the salmon module, containing 9 genes, also had a strong positive correlation with passage (Pearson correlation = 0.88, *p* value = 0.02). A negative correlation indicates that samples that have undergone 10 passages tend to have lower gene expression levels (i.e., downregulation) than those that have undergone 4 passages for genes in the yellow module. A positive correlation indicates that samples that have undergone 10 passages tend to have higher gene expression levels (i.e., upregulation) than those that have undergone 4 passages. Supplementary Figure 4 indicated that the yellow module and the salmon module had the highest gene significance values.

The scatter plot of gene significance vs. module membership in the yellow and salmon modules (Supplementary Figure 5) showed that GS and MM are highly correlated, implying that hub genes of the yellow and salmon modules also tend to be highly correlated with passage.

The WEB-based Gene SeT AnaLysis Toolkit showed that the yellow module was enriched in 59 KEGG pathways (e.g., Wnt signaling pathway, Axon guidance, MAPK signaling pathway, neurotrophin signaling pathway, endocytosis, TGF-beta signaling pathway, purine metabolism) and salmon module was enriched in one KEGG pathway (neurotrophin signaling pathway). Among the 742 genes in the yellow modules, 21 genes (Table 1) are in the Wnt pathway. All 9 genes (Table 2) in the salmon module are in the neurotrophin signaling pathway.

## 4. Conclusions

In this article, we took a network approach (WGCNA) to identify the gene network modules associated with the hDPSC passage. We identified two dental passage-associated modules (yellow module and salmon module) of genes. KEGG analysis showed that the yellow module was enriched in 59 KEGG pathways (including metabolic pathways, pathways in cancer, focal adhesion, Wnt signaling pathway, and MAPK signaling pathway). The salmon module was enriched in one KEGG signaling pathway (neurotrophin signaling pathway) in the salmon module [19].

The Wnt and MAPK pathways [20, 21] have all been related to DPSCs. The Wnt family is a group of signaling molecules that control a variety of developmental processes, including fate norms, proliferation, polarity, and cell migration as well as tooth renewal [22]. The gene SFRP2 in the Wnt pathway has been demonstrated to be able to enhance the adipogenic and neuronal differentiation potentials of dental pulp stem cells from apical papilla [23].

For the salmon module, some studies have reported that the gene neurotrophic factor neurotrophin- (NT-) 4 promotes the differentiation of dental epithelial cells and enhances the expression of enamel matrix genes [24].

**Figure 4:**
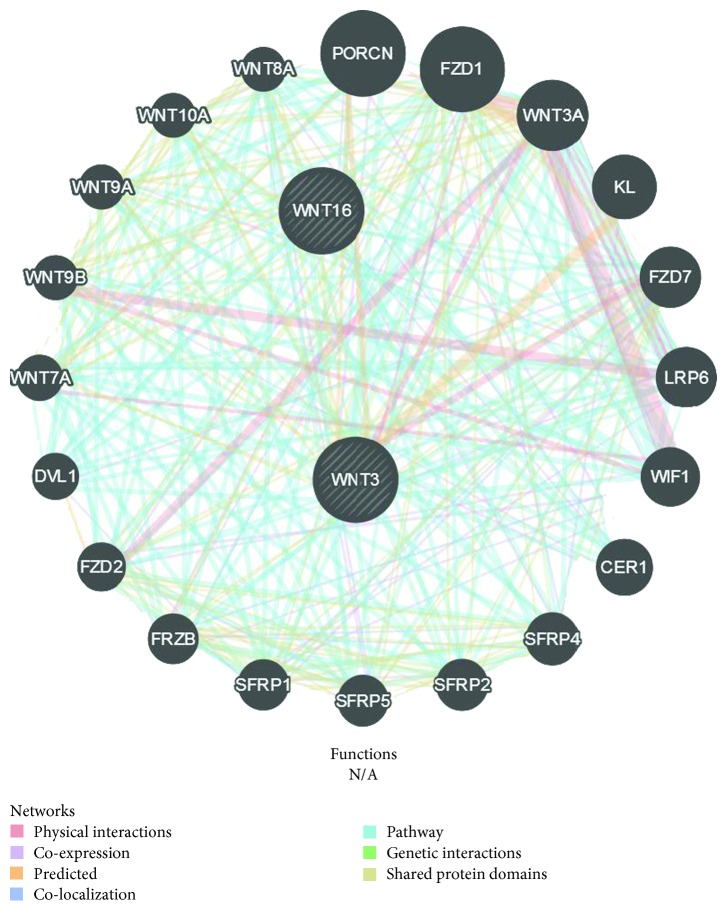
GeneMANIA analysis of yellow module.

The gene WNT16 identified by Takeda et al. [10] was not among the 21 genes in Supplementary Table 1 in Supplementary Materials. However, the yellow module contains the gene WNT3, which shares protein domain with WNT16 by GeneMANIA analysis [25] (Figure 4). Figure 4 contains 22 genes and there are 3 genes related to DPSC passage (SFRP2, WNT3, and LRP6) [26, 27].

There are a few limitations in this study. For example, the sample size in this study is small. However, we got two gene modules (yellow module and salmon module) significantly associated with hDPSC passage using the powerful network approach (WGCNA). There is no obvious hypothesis that is tested. Instead, the main goal of this article is to detect gene modules that are associated with the passage of hDPSCs. There are no independent datasets/experiments available yet to validate our results. We obtained some evidence from the GeneMANIA analysis that some genes in the yellow and salmon modules are related to the genes that are associated with hDPSCs. We identified 60 enriched pathways via the WebGestalt. Most of the 60 enriched pathways have not been related to the ability of differentiation of dental pulp stem cells yet. In the future, additional studies are needed to determine whether the genes or pathways that we identified as downregulated are responsible for the reduced regeneration capacity of the later stage passage and for validating the findings in this study. Another limitation is that the analysis is based on a preexisting database of the system. This precludes the unraveling of correlations that have not been found yet. However, the gene modules we identified help characterize the relationship among genes related to the passage of hDPSCs. Finally, in future researches, the effective involvement of the genes we identified should be further demonstrated in mechanistic and physiological studies, silencing or overexpressing each class of genes. Further investigation is warranted.

## Figures and Tables

**Table 1:** 21 genes enriched in the Wnt signaling pathway in the yellow module.

Index	User ID	Gene symbol	Entrez Gene	Ensembl
1	6423	SFRP2	6423	ENSG00000145423
2	1454	CSNK1E	1454	ENSG00000213923
3	144165	PRICKLE1	144165	ENSG00000139174
4	57216	VANGL2	57216	ENSG00000162738
5	5566	PRKACA	5566	ENSG00000072062
6	10725	NFAT5	10725	ENSG00000102908
7	23002	DAAM1	23002	ENSG00000100592
8	6934	TCF7L2	6934	ENSG00000148737
9	1387	CREBBP	1387	ENSG00000005339
10	3725	JUN	3725	ENSG00000177606
11	5529	PPP2R5E	5529	ENSG00000154001
12	4087	SMAD2	4087	ENSG00000175387
13	4775	NFATC3	4775	ENSG00000072736
14	6424	SFRP4	6424	ENSG00000106483
15	8312	AXIN1	8312	ENSG00000103126
16	5534	PPP3R1	5534	ENSG00000221823
17	6500	SKP1	6500	ENSG00000113558
18	7473	WNT3	7473	ENSG00000108379
19	1487	CTBP1	1487	ENSG00000159692
20	818	CAMK2G	818	ENSG00000148660
21	815	CAMK2A	815	ENSG00000070808

**Table 2:** Nine genes enriched in neurotrophic signaling pathways in salmon module.

Index	User ID	Gene symbol	Entrez Gene	Ensembl
1	817	CAMK2D	817	ENSG00000145349
2	2889	RAPGEF1	2889	ENSG00000107263
3	3654	IRAK1	3654	ENSG00000184216
4	10782	ZNF274	10782	ENSG00000171606
5	10971	YWHAQ	10971	ENSG00000134308
6	397	ARHGDIB	397	ENSG00000111348
7	3845	KRAS	3845	ENSG00000133703
8	2549	GAB1	2549	ENSG00000109458
9	27330	RPS6KA6	27330	ENSG00000072133

## Data Availability

The microarray data used to support the findings of this study have been deposited in the GEO repository. Please visit https://www.ncbi.nlm.nih.gov/geo/query/acc.cgi?acc=GSE10444 (GEO accession number: GSE10444).

## Supplementary Materials

Supplementary Materials Supplementary Figure 1: the plots of quantiles across arrays. (A) The original data. (B) The log2 transformed and quantile normalized expression data. Supplementary Figure 2: scatter plots of the top 2 principal components (pca plot). They were operated after log2 transformation and quantile normalization. (A) The pca plot with arrays colored with the crown-completed stage (2, 28, and 31 separately). (B) The pca plot with arrays colored with passage (4, 10). P4 samples and P10 samples are separated in the pca plot. (C) The pca plot with arrays colored with the combination of stage and passage. Supplementary Figure 3: hierarchical clustering of the six samples. Supplementary Figure 4: gene significance across modules. Supplementary Figure 5: scatter plots of gene significance (GS) for weight vs. module membership (MM) in the yellow and salmon modules. There is a highly significant correlation between GS and MM in these two modules. Supplementary Table 1: the enriched 58 KEGG pathways for the yellow module. Supplementary Table 2: the enriched 1 KEGG pathways for the salmon module. Supplementary Table 3: there were 660 genes upregulated at P10 in the yellow module. Supplementary Table 4: there were 7 genes upregulated at P10 in the salmon module.
