# Supplementary material for: An Investigation about Gene Modules Associated with hDPSC Differentiation for Adolescents
Source: Stem Cells Int. 2019 Apr 4;2019:8913287. doi: 10.1155/2019/8913287 (PMC6476005; doi:10.1155/2019/8913287)
Supplement: Supplementary Materials — Supplementary Figure 1: the plots of quantiles across arrays. (A) The original data. (B) The log2 transformed and quantile normalized expression data. Supplementary Figure 2: scatter plots of the top 2 principal components (pca plot). They were operated after log2 transformation and quantile normalization. (A) The pca plot with arrays colored with the crown-completed stage (2, 28, and 31 separately). (B) The pca plot with arrays colored with passage (4, 10). P4 samples and P10 samples are separated in the pca plot. (C) The pca plot with arrays colored with the combination of stage and passage. Supplementary Figure 3: hierarchical clustering of the six samples. Supplementary Figure 4: gene significance across modules. Supplementary Figure 5: scatter plots of gene significance (GS) for weight vs. module membership (MM) in the yellow and salmon modules. There is a highly significant correlation between GS and MM in these two modules. Supplementary Table 1: the enriched 58 KEGG pathways for the yellow module. Supplementary Table 2: the enriched 1 KEGG pathways for the salmon module. Supplementary Table 3: there were 660 genes upregulated at P10 in the yellow module. Supplementary Table 4: there were 7 genes upregulated at P10 in the salmon module. [file 8913287.f1.zip › Supplementary Table 3.pdf]

S3 Table. There were 660 genes up-regulated at P10 in the yellow module.

Gene Symbol	Gene Name	Entrez Gene
ADA	adenosine deaminase	100
LINC00476	long intergenic non-protein coding RNA 476	100128782
SLC25A21-AS1	SLC25A21 antisense RNA 1	100129794
HDAC6	histone deacetylase 6	10013
CKMT2-AS1	CKMT2 antisense RNA 1	100131067
TSTD1	thiosulfate sulfurtransferase like domain containing 1	100131187
MSC-AS1	MSC antisense RNA 1	100132891
BCL2L11	BCL2 like 11	10018
EML2-AS1	EML2 antisense RNA 1	100287177
SMKR1	small lysine rich protein 1	100287482
SLIT1-AS1	SLIT1 antisense RNA 1	100505540
LOC100505609	long intergenic non-protein coding RNA 2053	100505609
LINC01133	long intergenic non-protein coding RNA 1133	100505633
NAPA-AS1	NAPA antisense RNA 1	100505681
RPARP-AS1	RPARP antisense RNA 1	100505761
APTR	Alu-mediated CDKN1A/p21 transcriptional regulator (non-protein coding)	100505854
PTOV1-AS1	PTOV1 antisense RNA 1	100506033
LOC100506082	uncharacterized LOC100506082	100506082
LINC01503	long intergenic non-protein coding RNA 1503	100506119
LINC00867	long intergenic non-protein coding RNA 867	100506126
LARGE-AS1	LARGE antisense RNA 1	100506195
LOC100506274	uncharacterized LOC100506274	100506274
LOC100506302	uncharacterized LOC100506302	100506302
LOC100506498	uncharacterized LOC100506498	100506498
NRAV	negative regulator of antiviral response (non-protein coding)	100506668
ZNF865	zinc finger protein 865	100507290
LOC100507557	uncharacterized LOC100507557	100507557

LOC100508631	uncharacterized LOC100508631	100508631
TSPAN32	tetraspanin 32	10077
TMEM265	transmembrane protein 265	100862671
LOC100996419	uncharacterized LOC100996419	100996419
TSPAN2	tetraspanin 2	10100
LOC101927480	uncharacterized LOC101927480	101927480
ZNF529-AS1	ZNF529 antisense RNA 1	101927599
C5orf66-AS1	C5orf66 antisense RNA 1	101927953
LOC101928009	uncharacterized LOC101928009	101928009
TCONS_00029157	long intergenic non-protein coding RNA 1679	101928399
ZNF649-AS1	ZNF649 antisense RNA 1	101928571
LIPC-AS1	LIPC antisense RNA 1	101928694
LOC101928728	uncharacterized LOC101928728	101928728
LOC101929528	uncharacterized LOC101929528	101929528
LOC101929607	long intergenic non-protein coding RNA 1990	101929607
NBR2	neighbor of BRCA1 gene 2 (non-protein coding)	10230
RTN3	reticulon 3	10313
PCGF3	polycomb group ring finger 3	10336
EIF3M	eukaryotic translation initiation factor 3 subunit M	10480
NEBL	nebulette	10529
CDC42EP3	CDC42 effector protein 3	10602
RBCK1	RANBP2-type and C3HC4-type zinc finger containing 1	10616
TXNIP	thioredoxin interacting protein	10628
CNPY3	canopy FGF signaling regulator 3	10695
TRAF3IP2	TRAF3 interacting protein 2	10758
ARPP21	cAMP regulated phosphoprotein 21	10777
ZNF268	zinc finger protein 268	10795
CFTR	cystic fibrosis transmembrane conductance regulator	1080
TCERG1	transcription elongation regulator 1	10915
SRSF8	serine and arginine rich splicing factor 8	10929
METAP2	methionyl aminopeptidase 2	10988
SLC27A3	solute carrier family 27 member 3	11000
DUS4L	dihydrouridine synthase 4 like	11062

C10orf10	chromosome 10 open reading frame 10	11067
DIDO1	death inducer-obliterator 1	11083
NUDT4	nudix hydrolase 4	11163
ABCB8	ATP binding cassette subfamily B member 8	11194
IFT43	intraflagellar transport 43	112752
MFSD3	major facilitator superfamily domain containing 3	113655
SDSL	serine dehydratase like	113675
KLHDC7B	kelch domain containing 7B	113730
ZBED6CL	ZBED6 C-terminal like	113763
MBD6	methyl-CpG binding domain protein 6	114785
SLITRK1	SLIT and NTRK like family member 1	114798
PCMTD1	protein-L-isoaspartate (D-aspartate) O-methyltransferase domain containing 1	115294
GPR146	G protein-coupled receptor 146	115330
CYYR1	cysteine and tyrosine rich 1	116159
RBP7	retinol binding protein 7	116362
ZNF837	zinc finger protein 837	116412
RAB39B	RAB39B, member RAS oncogene family	116442
TOP1MT	topoisomerase (DNA) I, mitochondrial	116447
SH2D1B	SH2 domain containing 1B	117157
PRAP1	proline rich acidic protein 1	118471
COMTD1	catechol-O-methyltransferase domain containing 1	118881
ADD2	adducin 2	119
GSTO2	glutathione S-transferase omega 2	119391
ANAPC16	anaphase promoting complex subunit 16	119504
SFXN4	sideroflexin 4	119559
FAT3	FAT atypical cadherin 3	120114
TMEM45B	transmembrane protein 45B	120224
CYP2R1	cytochrome P450 family 2 subfamily R member 1	120227
RHEBL1	Ras homolog enriched in brain like 1	121268
C14orf28	chromosome 14 open reading frame	122525

	28	
ACOT4	acyl-CoA thioesterase 4	122970
MSI2	musashi RNA binding protein 2	124540
ZNF440	zinc finger protein 440	126070
SHE	Src homology 2 domain containing E	126669
CCSAP	centriole, cilia and spindle associated protein	126731
ERICH3	glutamate rich 3	127254
ZNF684	zinc finger protein 684	127396
C1orf131	chromosome 1 open reading frame 131	128061
SPATA17	spermatogenesis associated 17	128153
DRAM2	DNA damage regulated autophagy modulator 2	128338
COL4A2	collagen type IV alpha 2 chain	1284
SIRPD	signal regulatory protein delta	128646
COL5A1	collagen type V alpha 1 chain	1289
LYG1	lysozyme g1	129530
COL8A2	collagen type VIII alpha 2 chain	1296
COL11A2	collagen type XI alpha 2 chain	1302
PLEKHH2	pleckstrin homology, MyTH4 and FERM domain containing H2	130271
LYPD6	LY6/PLAUR domain containing 6	130574
GALM	galactose mutarotase	130589
TMEM182	transmembrane protein 182	130827
NADK2	NAD kinase 2, mitochondrial	133686
JMY	junction mediating and regulatory protein, p53 cofactor	133746
TMEM171	transmembrane protein 171	134285
IRAK1BP1	interleukin 1 receptor associated kinase 1 binding protein 1	134728
DPCR1	diffuse panbronchiolitis critical region 1	135656
NDUFAF6	NADH:ubiquinone oxidoreductase complex assembly factor 6	137682
C9orf116	chromosome 9 open reading frame 116	138162
FAM69B	family with sequence similarity 69 member B	138311
PTPDC1	protein tyrosine phosphatase domain containing 1	138639

RPP25L	ribonuclease P/MRP subunit p25 like	138716
TRIM69	tripartite motif containing 69	140691
UBE2F	ubiquitin conjugating enzyme E2 F (putative)	140739
SMCR8	Smith-Magenis syndrome chromosome region, candidate 8	140775
RPL10L	ribosomal protein L10 like	140801
NEURL2	neuralized E3 ubiquitin protein ligase 2	140825
R3HDML	R3H domain containing like	140902
PLEKHA7	pleckstrin homology domain containing A7	144100
PRICKLE1	prickle planar cell polarity protein 1	144165
ALG10B	ALG10B, alpha-1,2-glucosyltransferase	144245
ZNF664	zinc finger protein 664	144348
GLT1D1	glycosyltransferase 1 domain containing 1	144423
LACC1	laccase domain containing 1	144811
MIPOL1	mirror-image polydactyly 1	145282
PPP1R36	protein phosphatase 1 regulatory subunit 36	145376
LOC145694	uncharacterized LOC145694	145694
C2CD4A	C2 calcium dependent domain containing 4A	145741
C15orf65	chromosome 15 open reading frame 65	145788
TTBK2	tau tubulin kinase 2	146057
CMTM4	CKLF like MARVEL transmembrane domain containing 4	146223
RNF151	ring finger protein 151	146310
ZNF784	zinc finger protein 784	147808
CDC42EP5	CDC42 effector protein 5	148170
ZNF555	zinc finger protein 555	148254
MANEAL	mannosidase endo-alpha like	149175
RNF187	ring finger protein 187	149603
TTC30B	tetratricopeptide repeat domain 30B	150737
ZNF385B	zinc finger protein 385B	151126
METTL21A	methyltransferase like 21A	151194
WDSUB1	WD repeat, sterile alpha motif and U-box domain containing 1	151525

CCDC80	coiled-coil domain containing 80	151887
FGD5	FYVE, RhoGEF and PH domain containing 5	152273
CXADR	coxsackie virus and adenovirus receptor	1525
JAKMIP2-AS1	JAKMIP2 antisense RNA 1	153469
IRX2	iroquois homeobox 2	153572
TTC23L	tetratricopeptide repeat domain 23 like	153657
RNF217	ring finger protein 217	154214
CLEC2L	C-type lectin domain family 2 member L	154790
VPS37D	VPS37D, ESCRT-I subunit	155382
FBXO16	F-box protein 16	157574
SLC7A13	solute carrier family 7 member 13	157724
MCMDC2	minichromosome maintenance domain containing 2	157777
AK8	adenylate kinase 8	158067
KIAA1958	KIAA1958	158405
DYX1C1	dyslexia susceptibility 1 candidate 1	161582
SLFN5	schlafen family member 5	162394
ZNF780B	zinc finger protein 780B	163131
ACE	angiotensin I converting enzyme	1636
DCT	dopachrome tautomerase	1638
FREM3	FRAS1 related extracellular matrix 3	166752
SYNPO2	synaptopodin 2	171024
CLYBL	citrate lyase beta like	171425
DIAPH2	diaphanous related formin 2	1730
AGA	aspartylglucosaminidase	175
DNASE2	deoxyribonuclease 2, lysosomal	1777
DTNA	dystrobrevin alpha	1837
DUSP3	dual specificity phosphatase 3	1845
CYS1	cystin 1	192668
AGO3	argonaute 3, RISC catalytic component	192669
DNAH10	dynein axonemal heavy chain 10	196385
U2AF1L4	U2 small nuclear RNA auxiliary factor 1 like 4	199746
ZNF626	zinc finger protein 626	199777
TMEM201	transmembrane protein 201	199953

CC2D1B	coiled-coil and C2 domain containing 1B	200014
TMEM17	transmembrane protein 17	200728
ARL13B	ADP ribosylation factor like GTPase 13B	200894
LYRM9	LYR motif containing 9	201229
TMEM192	transmembrane protein 192	201931
STK32A	serine/threonine kinase 32A	202374
CCDC171	coiled-coil domain containing 171	203238
SUSD3	sushi domain containing 3	203328
EPHA1	EPH receptor A1	2041
EPHB1	EPH receptor B1	2047
SLC44A5	solute carrier family 44 member 5	204962
ERN1	endoplasmic reticulum to nucleus signaling 1	2081
UNC5B	unc-5 netrin receptor B	219699
TYSND1	trypsin domain containing 1	219743
TMEM218	transmembrane protein 218	219854
CFAP53	cilia and flagella associated protein 53	220136
8-Mar	membrane associated ring-CH-type finger 8	220972
LRRN4CL	LRRN4 C-terminal like	221091
AK9	adenylate kinase 9	221264
KIF6	kinesin family member 6	221458
JAZF1	JAZF zinc finger 1	221895
MTURN	maturin, neural progenitor differentiation regulator homolog	222166
FAM185A	family with sequence similarity 185 member A	222234
ATXN7L1	ataxin 7 like 1	222255
BEND7	BEN domain containing 7	222389
FAM83B	family with sequence similarity 83 member B	222584
ZNRF2	zinc and ring finger 2	223082
FHL1	four and a half LIM domains 1	2273
ITGA11	integrin subunit alpha 11	22801
AAK1	AP2 associated kinase 1	22848
CPEB3	cytoplasmic polyadenylation element binding protein 3	22849
MLXIP	MLX interacting protein	22877

ANKRD6	ankyrin repeat domain 6	22881
DAAM1	dishevelled associated activator of morphogenesis 1	23002
PEG10	paternally expressed 10	23089
ZNF423	zinc finger protein 423	23090
CLASP2	cytoplasmic linker associated protein 2	23122
KDM6B	lysine demethylase 6B	23135
CEP68	centrosomal protein 68	23177
PASK	PAS domain containing serine/threonine kinase	23178
MESDC2	mesoderm development candidate 2	23184
ACSBG1	acyl-CoA synthetase bubblegum family member 1	23205
RHOBTB2	Rho related BTB domain containing 2	23221
NUP210	nucleoporin 210	23225
EXOC6B	exocyst complex component 6B	23233
CAMTA1	calmodulin binding transcription activator 1	23261
CLASP1	cytoplasmic linker associated protein 1	23332
DOCK9	dedicator of cytokinesis 9	23348
KIAA0895	KIAA0895	23366
MED13L	mediator complex subunit 13 like	23389
ZDHHC17	zinc finger DHHC-type containing 17	23390
ABCA5	ATP binding cassette subfamily A member 5	23461
NPTXR	neuronal pentraxin receptor	23467
ZFYVE26	zinc finger FYVE-type containing 26	23503
TTC9	tetratricopeptide repeat domain 9	23508
SEZ6L	seizure related 6 homolog like	23544
ATP6V0A2	ATPase H+ transporting V0 subunit a2	23545
PSD4	pleckstrin and Sec7 domain containing 4	23550
CLDN15	claudin 15	24146
ZNF396	zinc finger protein 396	252884
STXBP4	syntaxin binding protein 4	252983
TCP11L2	t-complex 11 like 2	255394
FAM19A5	family with sequence similarity 19	25817

	member A5, C-C motif chemokine like	
BACE2	beta-site APP-cleaving enzyme 2	25825
PAMR1	peptidase domain containing associated with muscle regeneration 1	25891
ALS2CL	ALS2 C-terminal like	259173
TMIE	transmembrane inner ear	259236
SIN3A	SIN3 transcription regulator family member A	25942
ADGRA2	adhesion G protein-coupled receptor A2	25960
GGA1	golgi associated, gamma adaptin ear containing, ARF binding protein 1	26088
ZNF658	zinc finger protein 658	26149
RSL1D1	ribosomal L1 domain containing 1	26156
GIMAP2	GTPase, IMAP family member 2	26157
LRRC29	leucine rich repeat containing 29	26231
FBXL4	F-box and leucine rich repeat protein 4	26235
FBXO25	F-box protein 25	26260
SERGEF	secretion regulating guanine nucleotide exchange factor	26297
NR6A1	nuclear receptor subfamily 6 group A member 1	2649
GGCX	gamma-glutamyl carboxylase	2677
ST6GALNAC4	ST6 N-acetylgalactosaminide alpha-2,6-sialyltransferase 4	27090
ATP5S	ATP synthase, H+ transporting, mitochondrial Fo complex subunit s (factor B)	27109
SNX5	sorting nexin 5	27131
PCDH17	protocadherin 17	27253
LSM3	LSM3 homolog, U6 small nuclear RNA and mRNA degradation associated	27258
GPC1	glypican 1	2817
GPLD1	glycosylphosphatidylinositol specific phospholipase D1	2822
GPR1	G protein-coupled receptor 1	2825
ZNF311	zinc finger protein 311	282890
SBF2-AS1	SBF2 antisense RNA 1	283104
LOC283140	uncharacterized LOC283140	283140

PGM2L1	phosphoglucomutase 2 like 1	283209
HARBI1	harbinger transposase derived 1	283254
ADGRD1	adhesion G protein-coupled receptor D1	283383
FGF14-AS2	FGF14 antisense RNA 2	283481
LINC01146	long intergenic non-protein coding RNA 1146	283587
PRTG	protogenin	283659
LINC01270	long intergenic non-protein coding RNA 1270	284751
GPR27	G protein-coupled receptor 27	2850
CDH19	cadherin 19	28513
C3orf33	chromosome 3 open reading frame 33	285315
TCAIM	T-cell activation inhibitor, mitochondrial	285343
LOC285593	uncharacterized LOC285593	285593
KIAA0825	KIAA0825	285600
RNF180	ring finger protein 180	285671
PPIL6	peptidylprolyl isomerase like 6	285755
LY86-AS1	LY86 antisense RNA 1	285780
LINC00174	long intergenic non-protein coding RNA 174	285908
WEE2-AS1	WEE2 antisense RNA 1	285962
SLC26A4-AS1	SLC26A4 antisense RNA 1	286002
FAM86B3P	family with sequence similarity 86, member A pseudogene	286042
FAM83H	family with sequence similarity 83 member H	286077
ANK2	ankyrin 2	287
C11orf54	chromosome 11 open reading frame 54	28970
GRIK5	glutamate ionotropic receptor kainate type subunit 5	2901
ZBTB44	zinc finger and BTB domain containing 44	29068
CCDC113	coiled-coil domain containing 113	29070
ARHGAP35	Rho GTPase activating protein 35	2909
DMGDH	dimethylglycine dehydrogenase	29958
KCNIP3	potassium voltage-gated channel interacting protein 3	30818

EHD4	EH domain containing 4	30844
HLA-DQA1	major histocompatibility complex, class II, DQ alpha 1	3117
EIF4E3	eukaryotic translation initiation factor 4E family member 3	317649
ACACB	acetyl-CoA carboxylase beta	32
HOXA11	homeobox A11	3207
HOXB7	homeobox B7	3217
APBB2	amyloid beta precursor protein binding family B member 2	323
HSPA9	heat shock protein family A (Hsp70) member 9	3313
ICAM2	intercellular adhesion molecule 2	3384
C1orf228	chromosome 1 open reading frame 228	339541
BEX5	brain expressed X-linked 5	340542
NANOS1	nanos C2HC-type zinc finger 1	340719
SLC35F4	solute carrier family 35 member F4	341880
ZNF677	zinc finger protein 677	342926
IFIT2	interferon induced protein with tetratricopeptide repeats 2	3433
PAQR9	progestin and adipoQ receptor family member 9	344838
RSPH4A	radial spoke head 4 homolog A	345895
TUBB2B	tubulin beta 2B class IIb	347733
ZNF713	zinc finger protein 713	349075
NMNAT3	nicotinamide nucleotide adenylyltransferase 3	349565
IL6R	interleukin 6 receptor	3570
RFLNB	refilin B	359845
IL15	interleukin 15	3600
INSR	insulin receptor	3643
ITGAD	integrin subunit alpha D	3681
JARID2	jumonji and AT-rich interaction domain containing 2	3720
EFCAB5	EF-hand calcium binding domain 5	374786
ANKRD30B	ankyrin repeat domain 30B	374860
ATP9B	ATPase phospholipid transporting 9B (putative)	374868
TMEM205	transmembrane protein 205	374882

YJEFN3	YjeF N-terminal domain containing 3	374887
SPATA21	spermatogenesis associated 21	374955
AGRN	agrin	375790
SLC27A1	solute carrier family 27 member 1	376497
ZNF582-AS1	ZNF582 antisense RNA 1 (head to head)	386758
GUSBP2	glucuronidase, beta pseudogene 2	387036
SLC22A25	solute carrier family 22 member 25	387601
TMEM220	transmembrane protein 220	388335
SMIM1	small integral membrane protein 1 (Vel blood group)	388588
LINC01118	long intergenic non-protein coding RNA 1118	388948
KRT86	keratin 86	3892
LIG4	DNA ligase 4	3981
FADS3	fatty acid desaturase 3	3995
MEX3D	mex-3 RNA binding family member D	399664
CASC10	cancer susceptibility 10	399726
FLJ37035	uncharacterized LOC399821	399821
FAM174B	family with sequence similarity 174 member B	400451
FLJ22763	uncharacterized LOC401081	401081
OR2A20P	olfactory receptor family 2 subfamily A member 20 pseudogene	401428
WDFY3-AS2	WDFY3 antisense RNA 2	404201
MIR143	microRNA 143	406935
SMAD2	SMAD family member 2	4087
LCN10	lipocalin 10	414332
MCF2	MCF.2 cell line derived transforming sequence	4168
MDM2	MDM2 proto-oncogene	4193
MIA2	melanoma inhibitory activity 2	4253
MITF	melanogenesis associated transcription factor	4286
ATXN3	ataxin 3	4287
MOCS1	molybdenum cofactor synthesis 1	4337
MRE11	MRE11 homolog, double strand break repair nuclease	4361
UBL7-AS1	UBL7 antisense RNA 1 (head to head)	440288
LOC440934	uncharacterized LOC440934	440934

MTHFD2L	methylenetetrahydrofolate dehydrogenase (NADP+ dependent) 2-like	441024
ZFAS1	ZNFX1 antisense RNA 1	441951
MTCP1	mature T-cell proliferation 1	4515
NCAM1	neural cell adhesion molecule 1	4684
NDUFA10	NADH:ubiquinone oxidoreductase subunit A10	4705
NEDD9	neural precursor cell expressed, developmentally down-regulated 9	4739
NFE2L1	nuclear factor, erythroid 2 like 1	4779
NHS	NHS actin remodeling regulator	4810
NPAS2	neuronal PAS domain protein 2	4862
NTRK3	neurotrophic receptor tyrosine kinase 3	4916
ROR1	receptor tyrosine kinase like orphan receptor 1	4919
DFNB59	deafness, autosomal recessive 59	494513
OMD	osteomodulin	4958
TIFAB	TIFA inhibitor	497189
OSM	oncostatin M	5008
PAH	phenylalanine hydroxylase	5053
PAM	peptidylglycine alpha-amidating monooxygenase	5066
PAWR	pro-apoptotic WT1 regulator	5074
TMED5	transmembrane p24 trafficking protein 5	50999
AMDHD2	amidohydrolase domain containing 2	51005
SEPSECS	Sep (O-phosphoserine) tRNA:Sec (selenocysteine) tRNA synthase	51091
ING4	inhibitor of growth family member 4	51147
CERCAM	cerebral endothelial cell adhesion molecule	51148
MAPKAPK5-AS1	MAPKAPK5 antisense RNA 1	51275
ZNF117	zinc finger protein 117	51351
PDE1A	phosphodiesterase 1A	5136
TEX264	testis expressed 264	51368
CXXC5	CXXC finger protein 5	51523
HDAC7	histone deacetylase 7	51564
DPH5	diphthamide biosynthesis 5	51611

ERGIC3	ERGIC and golgi 3	51614
STYXL1	serine/threonine/tyrosine interacting like 1	51657
PECAM1	platelet and endothelial cell adhesion molecule 1	5175
ABCB1	ATP binding cassette subfamily B member 1	5243
PIP4K2A	phosphatidylinositol-5-phosphate 4-kinase type 2 alpha	5305
BCL11A	B-cell CLL/lymphoma 11A	53335
PLXNB1	plexin B1	5364
PMS1	PMS1 homolog 1, mismatch repair system component	5378
GPR85	G protein-coupled receptor 85	54329
FAM134B	family with sequence similarity 134 member B	54463
RNF216	ring finger protein 216	54476
PLEKHA5	pleckstrin homology domain containing A5	54477
MIER2	MIER family member 2	54531
MRPL50	mitochondrial ribosomal protein L50	54534
NECAB2	N-terminal EF-hand calcium binding protein 2	54550
SPATA6	spermatogenesis associated 6	54558
GNB1L	G protein subunit beta 1 like	54584
LZTFL1	leucine zipper transcription factor like 1	54585
EQTN	equatorin	54586
ZNF853	zinc finger protein 853	54753
KLHL24	kelch like family member 24	54800
GIN1	gypsy retrotransposon integrase 1	54826
PGPEP1	pyroglutamyl-peptidase I	54858
FNBP1L	formin binding protein 1 like	54874
WHSC1L1	nuclear receptor binding SET domain protein 3	54904
EXD3	exonuclease 3'-5' domain containing 3	54932
RNF125	ring finger protein 125	54941
ULK4	unc-51 like kinase 4	54986
KIF26B	kinesin family member 26B	55083
ANO1	anoctamin 1	55107

RAVER2	ribonucleoprotein, PTB binding 2	55225
QRSL1	glutaminyl-tRNA synthase (glutamine-hydrolyzing)-like 1	55278
SYNJ2BP	synaptojanin 2 binding protein	55333
NIPSNAP3B	nipsnap homolog 3B	55335
YOD1	YOD1 deubiquitinase	55432
PRIM2	primase (DNA) subunit 2	5558
KIF27	kinesin family member 27	55582
PRKAB2	protein kinase AMP-activated non-catalytic subunit beta 2	5565
SLC48A1	solute carrier family 48 member 1	55652
BCAS4	breast carcinoma amplified sequence 4	55653
11-Sep	septin 11	55752
TTC17	tetratricopeptide repeat domain 17	55761
ZC4H2	zinc finger C4H2-type containing	55906
BAIAP2L1	BAI1 associated protein 2 like 1	55971
MYDGF	myeloid derived growth factor	56005
PCDHGC4	protocadherin gamma subfamily C, 4	56098
BEX4	brain expressed X-linked 4	56271
CCL28	C-C motif chemokine ligand 28	56477
PSEN2	presenilin 2	5664
B2M	beta-2-microglobulin	567
PSMA3	proteasome subunit alpha 3	5684
MEIS3	Meis homeobox 3	56917
LHX9	LIM homeobox 9	56956
C14orf132	chromosome 14 open reading frame 132	56967
STOX2	storkhead box 2	56977
PRDM11	PR/SET domain 11	56981
ADAMTS9	ADAM metallopeptidase with thrombospondin type 1 motif 9	56999
CABP4	calcium binding protein 4	57010
SLC17A7	solute carrier family 17 member 7	57030
VANGL2	VANGL planar cell polarity protein 2	57216
LYRM2	LYR motif containing 2	57226
PTGFRN	prostaglandin F2 receptor inhibitor	5738
MIF4GD	MIF4G domain containing	57409
PPM1H	protein phosphatase, Mg2+/Mn2+	57460

	dependent 1H	
ISY1	ISY1 splicing factor homolog	57461
NLGN4X	neuroligin 4, X-linked	57502
MTUS1	microtubule associated tumor suppressor 1	57509
CGN	cingulin	57530
KIAA1328	KIAA1328	57536
SORCS2	sortilin related VPS10 domain containing receptor 2	57537
WDR35	WD repeat domain 35	57539
CCDC191	coiled-coil domain containing 191	57577
KIAA1549	KIAA1549	57670
ZNF250	zinc finger protein 250	58500
RALA	RAS like proto-oncogene A	5898
ENPP5	ectonucleotide pyrophosphatase/phosphodiesterase 5 (putative)	59084
RXFP1	relaxin/insulin like family peptide receptor 1	59350
RBM4	RNA binding motif protein 4	5936
RFX3	regulatory factor X3	5991
RGS12	regulator of G-protein signaling 12	6002
RHEB	Ras homolog enriched in brain	6009
GPBP1L1	GC-rich promoter binding protein 1 like 1	60313
ELOVL5	ELOVL fatty acid elongase 5	60481
GAS5	growth arrest specific 5 (non-protein coding)	60674
RPL37	ribosomal protein L37	6167
RPS27A	ribosomal protein S27a	6233
RPS29	ribosomal protein S29	6235
SAT1	spermidine/spermine N1-acetyltransferase 1	6303
SBF1	SET binding factor 1	6305
SDC2	syndecan 2	6383
ANKEF1	ankyrin repeat and EF-hand domain containing 1	63926
SEC14L1	SEC14 like lipid binding 1	6397
PRDM16	PR/SET domain 16	63976
SEMG2	semenogelin II	6407

EPB41L4A	erythrocyte membrane protein band 4.1 like 4A	64097
C10orf54	V-set immunoregulatory receptor	64115
DNAJC1	DnaJ heat shock protein family (Hsp40) member C1	64215
SFRP2	secreted frizzled related protein 2	6423
SFRP4	secreted frizzled related protein 4	6424
CCT6P3	chaperonin containing TCP1 subunit 6 pseudogene 3	643180
AZI2	5-azacytidine induced 2	64343
NOM1	nucleolar protein with MIF4G domain 1	64434
LOC645166	lymphocyte-specific protein 1 pseudogene	645166
GPR135	G protein-coupled receptor 135	64582
BNC1	basonuclin 1	646
HRCT1	histidine rich carboxyl terminus 1	646962
SHMT2	serine hydroxymethyltransferase 2	6472
RMND5A	required for meiotic nuclear division 5 homolog A	64795
BCL11B	B-cell CLL/lymphoma 11B	64919
STAG3L4	stromal antigen 3-like 4 (pseudogene)	64940
RAPH1	Ras association (RalGDS/AF-6) and pleckstrin homology domains 1	65059
WNK4	WNK lysine deficient protein kinase 4	65266
SLC7A4	solute carrier family 7 member 4	6545
SLC19A1	solute carrier family 19 member 1	6573
KCTD14	potassium channel tetramerization domain containing 14	65987
SRPK2	SRSF protein kinase 2	6733
SSTR5	somatostatin receptor 5	6755
SUPV3L1	Suv3 like RNA helicase	6832
SYCP1	synaptonemal complex protein 1	6847
TAF4B	TATA-box binding protein associated factor 4b	6875
TCEA2	transcription elongation factor A2	6919
TCF7L2	transcription factor 7 like 2	6934
TERT	telomerase reverse transcriptase	7015
TFDP2	transcription factor Dp-2	7029

TIMP3	TIMP metallopeptidase inhibitor 3	7078
TLE4	transducin like enhancer of split 4	7091
TLR4	toll like receptor 4	7099
TM7SF2	transmembrane 7 superfamily member 2	7108
TPD52	tumor protein D52	7163
LINC01137	long intergenic non-protein coding RNA 1137	728431
CCDC169	coiled-coil domain containing 169	728591
ZNF736	zinc finger protein 736	728927
LOC729083	long intergenic non-protein coding RNA 2158	729083
TNFRSF4	TNF receptor superfamily member 4	7293
CORO2A	coronin 2A	7464
ZXDA	zinc finger, X-linked, duplicated A	7789
LAPTM5	lysosomal protein transmembrane 5	7805
FAM134A	family with sequence similarity 134 member A	79137
MMP28	matrix metallopeptidase 28	79148
ALDH5A1	aldehyde dehydrogenase 5 family member A1	7915
HSD17B8	hydroxysteroid 17-beta dehydrogenase 8	7923
BHLHE41	basic helix-loop-helix family member e41	79365
SLC52A2	solute carrier family 52 member 2	79581
TMEM231	transmembrane protein 231	79583
CALCA	calcitonin related polypeptide alpha	796
RNASEH2B	ribonuclease H2 subunit B	79621
EFCAB1	EF-hand calcium binding domain 1	79645
SAP30L	SAP30 like	79685
NSUN7	NOP2/Sun RNA methyltransferase family member 7	79730
WDR78	WD repeat domain 78	79819
SETD6	SET domain containing 6	79918
UBXN8	UBX domain protein 8	7993
MYCT1	myc target 1	80177
RUBCNL	RUN and cysteine rich domain containing beclin 1 interacting protein like	80183

TMEM254	transmembrane protein 254	80195
HKDC1	hexokinase domain containing 1	80201
ALPK1	alpha kinase 1	80216
CEP70	centrosomal protein 70	80321
PABPC1L	poly(A) binding protein cytoplasmic 1 like	80336
WDR61	WD repeat domain 61	80349
SPX	spexin hormone	80763
CLPTM1L	CLPTM1 like	81037
ANP32A	acidic nuclear phosphoprotein 32 family member A	8125
HM13	histocompatibility minor 13	81502
GDPD5	glycerophosphodiester phosphodiesterase domain containing 5	81544
TDRD3	tudor domain containing 3	81550
CANX	calnexin	821
CAPS	calcyphosine	828
CAST	calpastatin	831
AXIN1	axin 1	8312
PITPNM3	PITPNM family member 3	83394
INO80B	INO80 complex subunit B	83444
ABHD11	abhydrolase domain containing 11	83451
SH3BGRL2	SH3 domain binding glutamate rich protein like 2	83699
SETDB2	SET domain bifurcated 2	83852
SPATA9	spermatogenesis associated 9	83890
RASSF4	Ras association domain family member 4	83937
CCDC8	coiled-coil domain containing 8	83987
FAM161A	family with sequence similarity 161 member A	84140
KAT8	lysine acetyltransferase 8	84148
LOXL4	lysyl oxidase like 4	84171
MFSD7	major facilitator superfamily domain containing 7	84179
ALKBH7	alkB homolog 7	84266
EFCAB2	EF-hand calcium binding domain 2	84288
FAM213A	family with sequence similarity 213 member A	84293

CCDC115	coiled-coil domain containing 115	84317
ZBED3	zinc finger BED-type containing 3	84327
CYSTM1	cysteine rich transmembrane module containing 1	84418
ABLIM2	actin binding LIM protein family member 2	84448
ZMAT1	zinc finger matrin-type 1	84460
PLPP5	phospholipid phosphatase 5	84513
FAXC	failed axon connections homolog	84553
SLITRK2	SLIT and NTRK like family member 2	84631
ACCS	1-aminocyclopropane-1-carboxylate synthase homolog (inactive)	84680
PLEKHA8	pleckstrin homology domain containing A8	84725
USP30	ubiquitin specific peptidase 30	84749
CDC42BPA	CDC42 binding protein kinase alpha	8476
HIRIP3	HIRA interacting protein 3	8479
IL17RC	interleukin 17 receptor C	84818
SEMA7A	semaphorin 7A (John Milton Hagen blood group)	8482
RPUSD4	RNA pseudouridylate synthase domain containing 4	84881
POMGNT2	protein O-linked mannose N-acetylglucosaminyltransferase 2 (beta 1,4-)	84892
ALG10	ALG10, alpha-1,2-glucosyltransferase	84920
TMEM209	transmembrane protein 209	84928
ZNRF1	zinc and ring finger 1	84937
CGNL1	cingulin like 1	84952
SYTL1	synaptotagmin like 1	84958
FBXL20	F-box and leucine rich repeat protein 20	84961
AJUBA	ajuba LIM protein	84962
PNMA6A	paraneoplastic Ma antigen family member 6A	84968
PHYKPL	5-phosphohydroxy-L-lysine phospho-lyase	85007
RHPN2	rhophilin Rho GTPase binding protein 2	85415
ZNF518B	zinc finger protein 518B	85460

PDE8B	phosphodiesterase 8B	8622
HSD17B6	hydroxysteroid 17-beta dehydrogenase 6	8630
RNASET2	ribonuclease T2	8635
NCOA1	nuclear receptor coactivator 1	8648
ADAM15	ADAM metallopeptidase domain 15	8751
MPDZ	multiple PDZ domain crumbs cell polarity complex component	8777
PEX11A	peroxisomal biogenesis factor 11 alpha	8800
INPP4B	inositol polyphosphate-4-phosphatase type II B	8821
NRP2	neuropilin 2	8828
PAPLN	papilin, proteoglycan like sulfated glycoprotein	89932
GLB1L2	galactosidase beta 1 like 2	89944
UBE3D	ubiquitin protein ligase E3D	90025
PHLDB2	pleckstrin homology like domain family B member 2	90102
C9orf69	chromosome 9 open reading frame 69	90120
FRMD7	FERM domain containing 7	90167
SYS1	SYS1, golgi trafficking protein	90196
ZNF468	zinc finger protein 468	90333
FAM110B	family with sequence similarity 110 member B	90362
BMF	Bcl2 modifying factor	90427
YIF1B	Yip1 interacting factor homolog B, membrane trafficking protein	90522
N4BP2L1	NEDD4 binding protein 2 like 1	90634
CEP95	centrosomal protein 95	90799
ZNF598	zinc finger protein 598	90850
ADCK2	aarF domain containing kinase 2	90956
GUSBP11	glucuronidase, beta pseudogene 11	91316
DYRK1B	dual specificity tyrosine phosphorylation regulated kinase 1B	9149
SMDT1	single-pass membrane protein with aspartate rich tail 1	91689
ALKBH8	alkB homolog 8, tRNA methyltransferase	91801

C11orf52	chromosome 11 open reading frame 52	91894
TTC30A	tetratricopeptide repeat domain 30A	92104
ATP6AP1L	ATPase H+ transporting accessory protein 1 like	92270
CHMP4C	charged multivesicular body protein 4C	92421
HELB	DNA helicase B	92797
NT5C1B	5'-nucleotidase, cytosolic IB	93034
DMKN	dermokine	93099
PIGM	phosphatidylinositol glycan anchor biosynthesis class M	93183
IGSF8	immunoglobulin superfamily member 8	93185
MVB12A	multivesicular body subunit 12A	93343
SDR42E1	short chain dehydrogenase/reductase family 42E, member 1	93517
TRMT10A	tRNA methyltransferase 10A	93587
RAB28	RAB28, member RAS oncogene family	9364
RECQL5	RecQ like helicase 5	9400
EPSTI1	epithelial stromal interaction 1	94240
TP53INP1	tumor protein p53 inducible nuclear protein 1	94241
PGS1	phosphatidylglycerophosphate synthase 1	9489
CD40	CD40 molecule	958
N4BP1	NEDD4 binding protein 1	9683
VGLL4	vestigial like family member 4	9686
CEP57	centrosomal protein 57	9702
USP6NL	USP6 N-terminal like	9712
ARHGAP32	Rho GTPase activating protein 32	9743
ZFYVE16	zinc finger FYVE-type containing 16	9765
MRPL19	mitochondrial ribosomal protein L19	9801
HEPH	hephaestin	9843
