# Supplementary material for: An Investigation about Gene Modules Associated with hDPSC Differentiation for Adolescents
Source: Stem Cells Int. 2019 Apr 4;2019:8913287. doi: 10.1155/2019/8913287 (PMC6476005; doi:10.1155/2019/8913287)
Supplement: Supplementary Materials — Supplementary Figure 1: the plots of quantiles across arrays. (A) The original data. (B) The log2 transformed and quantile normalized expression data. Supplementary Figure 2: scatter plots of the top 2 principal components (pca plot). They were operated after log2 transformation and quantile normalization. (A) The pca plot with arrays colored with the crown-completed stage (2, 28, and 31 separately). (B) The pca plot with arrays colored with passage (4, 10). P4 samples and P10 samples are separated in the pca plot. (C) The pca plot with arrays colored with the combination of stage and passage. Supplementary Figure 3: hierarchical clustering of the six samples. Supplementary Figure 4: gene significance across modules. Supplementary Figure 5: scatter plots of gene significance (GS) for weight vs. module membership (MM) in the yellow and salmon modules. There is a highly significant correlation between GS and MM in these two modules. Supplementary Table 1: the enriched 58 KEGG pathways for the yellow module. Supplementary Table 2: the enriched 1 KEGG pathways for the salmon module. Supplementary Table 3: there were 660 genes upregulated at P10 in the yellow module. Supplementary Table 4: there were 7 genes upregulated at P10 in the salmon module. [file 8913287.f1.zip › Supplementary Table 1.pdf]

S1 Table. The enriched 58 KEGG pathways for the yellow module.

PathwayName	#Gene	Statistics
Metabolic pathways	86	C=1130;O=86;E=44.62;R=1.93;rawP=6.66e-09;adjP=1.15e-06
Pathways in cancer	34	C=326;O=34;E=12.87;R=2.64;rawP=3.13e-07;adjP=2.20e-05
Focal adhesion	25	C=200;O=25;E=7.90;R=3.17;rawP=4.03e-07;adjP=2.20e-05
Wnt signaling pathway	21	C=150;O=21;E=5.92;R=3.55;rawP=5.12e-07;adjP=2.20e-05
ECM-receptor interaction	14	C=85;O=14;E=3.36;R=4.17;rawP=5.84e-06;adjP=0.0001
Hypertrophic cardiomyopathy (HCM)	14	C=83;O=14;E=3.28;R=4.27;rawP=4.37e-06;adjP=0.0001
Axon guidance	18	C=129;O=18;E=5.09;R=3.53;rawP=3.42e-06;adjP=0.0001
Phagosome	19	C=153;O=19;E=6.04;R=3.14;rawP=1.05e-05;adjP=0.0002
Regulation of actin cytoskeleton	23	C=213;O=23;E=8.41;R=2.73;rawP=1.38e-05;adjP=0.0003
Cell adhesion molecules (CAMs)	17	C=133;O=17;E=5.25;R=3.24;rawP=2.06e-05;adjP=0.0004
Insulin signaling pathway	17	C=138;O=17;E=5.45;R=3.12;rawP=3.34e-05;adjP=0.0004
MAPK signaling pathway	26	C=268;O=26;E=10.58;R=2.46;rawP=2.62e-05;adjP=0.0004
Adherens junction	12	C=73;O=12;E=2.88;R=4.16;rawP=2.73e-05;adjP=0.0004
Protein processing in endoplasmic reticulum	19	C=165;O=19;E=6.52;R=2.92;rawP=3.08e-05;adjP=0.0004
Arrhythmogenic right ventricular cardiomyopathy (ARVC)	12	C=74;O=12;E=2.92;R=4.11;rawP=3.14e-05;adjP=0.0004
Dilated cardiomyopathy	13	C=90;O=13;E=3.55;R=3.66;rawP=5.25e-05;adjP=0.0006
Small cell lung cancer	12	C=85;O=12;E=3.36;R=3.58;rawP=0.0001;adjP=0.0010
Neurotrophin signaling pathway	15	C=127;O=15;E=5.01;R=2.99;rawP=0.0002;adjP=0.0019

Endocytosis	19	C=201;O=19;E=7.94;R=2.39;rawP=0.0004;adjP=0.0034
Viral myocarditis	10	C=70;O=10;E=2.76;R=3.62;rawP=0.0004;adjP=0.0034
TGF-beta signaling pathway	11	C=84;O=11;E=3.32;R=3.32;rawP=0.0005;adjP=0.0041
ErbB signaling pathway	11	C=87;O=11;E=3.44;R=3.20;rawP=0.0006;adjP=0.0045
N-Glycan biosynthesis	8	C=49;O=8;E=1.93;R=4.13;rawP=0.0006;adjP=0.0045
Circadian rhythm - mammal	5	C=22;O=5;E=0.87;R=5.76;rawP=0.0014;adjP=0.0100
Pathogenic Escherichia coli infection	8	C=56;O=8;E=2.21;R=3.62;rawP=0.0015;adjP=0.0103
Renal cell carcinoma	9	C=70;O=9;E=2.76;R=3.26;rawP=0.0017;adjP=0.0104
Vasopressin-regulated water reabsorption	7	C=44;O=7;E=1.74;R=4.03;rawP=0.0016;adjP=0.0104
Long-term potentiation	9	C=70;O=9;E=2.76;R=3.26;rawP=0.0017;adjP=0.0104
Bile secretion	9	C=71;O=9;E=2.80;R=3.21;rawP=0.0019;adjP=0.0109
Melanoma	9	C=71;O=9;E=2.80;R=3.21;rawP=0.0019;adjP=0.0109
Purine metabolism	15	C=162;O=15;E=6.40;R=2.34;rawP=0.0020;adjP=0.0111
Notch signaling pathway	7	C=47;O=7;E=1.86;R=3.77;rawP=0.0023;adjP=0.0124
Prostate cancer	10	C=89;O=10;E=3.51;R=2.85;rawP=0.0027;adjP=0.0141
Gap junction	10	C=90;O=10;E=3.55;R=2.81;rawP=0.0029;adjP=0.0147
Endometrial cancer	7	C=52;O=7;E=2.05;R=3.41;rawP=0.0042;adjP=0.0195
Glioma	8	C=65;O=8;E=2.57;R=3.12;rawP=0.0040;adjP=0.0195
mTOR signaling pathway	7	C=52;O=7;E=2.05;R=3.41;rawP=0.0042;adjP=0.0195
One carbon pool by folate	4	C=18;O=4;E=0.71;R=5.63;rawP=0.0048;adjP=0.0217
RNA polymerase	5	C=29;O=5;E=1.15;R=4.37;rawP=0.0051;adjP=0.0225
Adipocytokine	8	C=68;O=8;E=2.69;R=2.98;rawP=0.0053

signaling pathway		;adjP=0.0228
Bacterial invasion of epithelial cells	8	C=70;O=8;E=2.76;R=2.89;rawP=0.0063;adjP=0.0264
Melanogenesis	10	C=101;O=10;E=3.99;R=2.51;rawP=0.0067;adjP=0.0274
ABC transporters	6	C=44;O=6;E=1.74;R=3.45;rawP=0.0073;adjP=0.0277
Inositol phosphate metabolism	7	C=57;O=7;E=2.25;R=3.11;rawP=0.0070;adjP=0.0277
Ubiquitin mediated proteolysis	12	C=135;O=12;E=5.33;R=2.25;rawP=0.0074;adjP=0.0277
Leishmaniasis	8	C=72;O=8;E=2.84;R=2.81;rawP=0.0074;adjP=0.0277
Gastric acid secretion	8	C=74;O=8;E=2.92;R=2.74;rawP=0.0087;adjP=0.0318
Amoebiasis	10	C=106;O=10;E=4.19;R=2.39;rawP=0.0093;adjP=0.0333
Antigen processing and presentation	8	C=76;O=8;E=3.00;R=2.67;rawP=0.0102;adjP=0.0358
Ribosome	9	C=92;O=9;E=3.63;R=2.48;rawP=0.0105;adjP=0.0361
Intestinal immune network for IgA production	6	C=48;O=6;E=1.90;R=3.17;rawP=0.0112;adjP=0.0363
Calcium signaling pathway	14	C=177;O=14;E=6.99;R=2.00;rawP=0.0109;adjP=0.0363
Type II diabetes mellitus	6	C=48;O=6;E=1.90;R=3.17;rawP=0.0112;adjP=0.0363
Spliceosome	11	C=127;O=11;E=5.01;R=2.19;rawP=0.0121;adjP=0.0385
Glycerophospholipid metabolism	8	C=80;O=8;E=3.16;R=2.53;rawP=0.0136;adjP=0.0425
Butirosin and neomycin biosynthesis	2	C=5;O=2;E=0.20;R=10.13;rawP=0.0144;adjP=0.0442
Malaria	6	C=51;O=6;E=2.01;R=2.98;rawP=0.0148;adjP=0.0447
Glycosylphosphatidylinositol(GPI)-anchor biosynthesis	4	C=25;O=4;E=0.99;R=4.05;rawP=0.0158;adjP=0.0469
Autoimmune thyroid disease	6	C=52;O=6;E=2.05;R=2.92;rawP=0.0162;adjP=0.0472
