# Supplementary figures and images for: An Investigation about Gene Modules Associated with hDPSC Differentiation for Adolescents
Source: Stem Cells Int. 2019 Apr 4;2019:8913287. doi: 10.1155/2019/8913287 (PMC6476005; doi:10.1155/2019/8913287)

S3 Fig. Hierarchical clustering of the six samples.

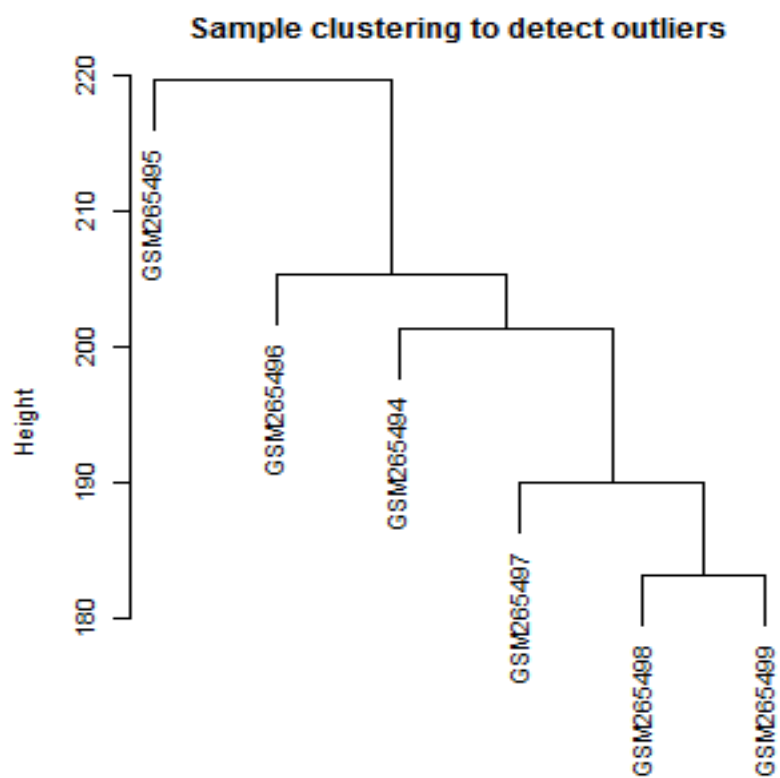

Supplement: Supplementary Materials — Supplementary Figure 1: the plots of quantiles across arrays. (A) The original data. (B) The log2 transformed and quantile normalized expression data. Supplementary Figure 2: scatter plots of the top 2 principal components (pca plot). They were operated after log2 transformation and quantile normalization. (A) The pca plot with arrays colored with the crown-completed stage (2, 28, and 31 separately). (B) The pca plot with arrays colored with passage (4, 10). P4 samples and P10 samples are separated in the pca plot. (C) The pca plot with arrays colored with the combination of stage and passage. Supplementary Figure 3: hierarchical clustering of the six samples. Supplementary Figure 4: gene significance across modules. Supplementary Figure 5: scatter plots of gene significance (GS) for weight vs. module membership (MM) in the yellow and salmon modules. There is a highly significant correlation between GS and MM in these two modules. Supplementary Table 1: the enriched 58 KEGG pathways for the yellow module. Supplementary Table 2: the enriched 1 KEGG pathways for the salmon module. Supplementary Table 3: there were 660 genes upregulated at P10 in the yellow module. Supplementary Table 4: there were 7 genes upregulated at P10 in the salmon module. [file 8913287.f1.zip › Supplementary Figure 3.pdf]

**S4 Fig. Gene significance across modules.**

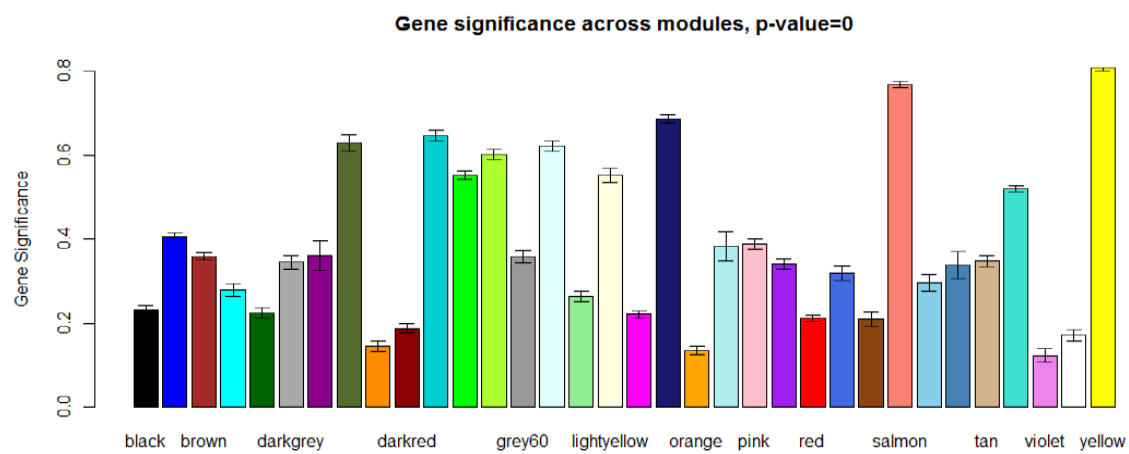

Supplement: Supplementary Materials — Supplementary Figure 1: the plots of quantiles across arrays. (A) The original data. (B) The log2 transformed and quantile normalized expression data. Supplementary Figure 2: scatter plots of the top 2 principal components (pca plot). They were operated after log2 transformation and quantile normalization. (A) The pca plot with arrays colored with the crown-completed stage (2, 28, and 31 separately). (B) The pca plot with arrays colored with passage (4, 10). P4 samples and P10 samples are separated in the pca plot. (C) The pca plot with arrays colored with the combination of stage and passage. Supplementary Figure 3: hierarchical clustering of the six samples. Supplementary Figure 4: gene significance across modules. Supplementary Figure 5: scatter plots of gene significance (GS) for weight vs. module membership (MM) in the yellow and salmon modules. There is a highly significant correlation between GS and MM in these two modules. Supplementary Table 1: the enriched 58 KEGG pathways for the yellow module. Supplementary Table 2: the enriched 1 KEGG pathways for the salmon module. Supplementary Table 3: there were 660 genes upregulated at P10 in the yellow module. Supplementary Table 4: there were 7 genes upregulated at P10 in the salmon module. [file 8913287.f1.zip › Supplementary Figure 4.pdf]
